# Femurkopfreduktionsosteotomie zur Verbesserung des femoroazetabulären Containments bei Morbus Perthes

**DOI:** 10.1007/s00064-022-00779-2

**Published:** 2022-07-21

**Authors:** Theddy Slongo, Kai Ziebarth

**Affiliations:** Abteilung Kinderorthopädie, Kinderchirurgische Universitätsklinik, Inselspital Bern, Freiburgstr., 3010 Bern, Schweiz

**Keywords:** Asphärizität Hüftkopf, Inkongruenz, Containment-Verlust, Hüftkopfreduktion, Chirurgische Hüftluxation, Femoral head asphericity, Incongruence, Loss of containment, Femoral head reduction, Surgical hip dislocation

## Abstract

**Operationsziel:**

Wiederherstellung der Hüftkongruenz und des Containments durch zentrale Hüftkopfresektion/Reduktion über eine erweiterte chirurgische Hüftluxation unter Erhaltung/Respektierung der Hüftkopfdurchblutung. Eine gleichzeitige oder spätere Reorientierung des Acetabulums durch Triple-Osteotomie oder PAO bei instabiler Hüfte kann in speziellen Fällen notwendig werden.

**Indikationen:**

Die Indikation zur chirurgischen Hüftkopfreduktion ist praktisch unabhängig vom Alter bei jeglicher inkongruenten, asphärischen Hüftkopfsituation mit „hinged abduction“ (zu erwartende Endsituation wie Stulberg IV und V) gegeben. Dies kann noch bei aktivem wie auch bei bereits ausgeheiltem Morbus Perthes der Fall sein. Zudem kann bei einer Stulberg-V-Situation selbst im adulten Alter noch eine Verbesserung erziel werden. Nach Resektion muss noch ein tragfähiger Resthüftkopf vorhanden bleiben, d. h. mindestens noch 50 % des ausgeweiteten Hüftkopfes. Die dafür beste Planung erfolgt in der „vergleichenden“ 3‑D-Rekonstruktion.

**Kontraindikationen:**

Völlig zerstörter Knorpel oder Hüftkopf.

**Operationstechnik:**

Identisches Operationsvorgehen wie für die klassische chirurgische Hüftluxation. Präparation der retinakulären Flaps. Unter Respektierung und in Kenntnis der Gefäßversorgung Spaltung des Hüftkopfes gemäß dem zu entfernenden, nekrotischen Kopfanteil. Bildung eines möglichst sphärischen Hüftkopfes und Verschraubung der beiden Kopfanteile auf Schenkelhalsniveau. Distalisation und Fixierung des großen Trochanters. Je nach Kongruenz und Stabilität des Hüftkopfs in der Hüftpfanne kann eine primäre oder sekundäre Triple-OT oder PAO notwendig werden.

**Weiterbehandlung:**

Die intraoperative Stabilität des Femurkopfes im Acetabulum muss erzielt worden sein, um eine beckengipsfreie, funktionelle Nachbehandlung zu gewährleisten: Stockentlastung mit Bodenkontakt ist erlaubt; keine aktive Rotation; Flexion aktiv und passiv bis 90 Grad ist erlaubt; vorerst keine spezifische Physiotherapie; je nach Heilungsverlauf sind diese Maßnahmen 8 bis 10 Wochen einzuhalten.

**Ergebnisse:**

Gemäß unseren publizierten Nachuntersuchungen (aktuell 21 Jahre) sehen wir bei technisch korrekt durchgeführter Operation und korrekter Indikation sowie adäquater Nachbehandlung durchwegs gute Ergebnisse. Nekrosen des reduzierten Hüftkopfs haben wir nie beobachtet. Alle gespaltenen Hüftköpfe, respektive Schenkelhälse sind primär geheilt.

## Abstract (en)

**Objective:**

Restoration of hip congruence and containment through central femoral head resection/reduction via an extended surgical hip dislocation, while maintaining/respecting the femoral head blood flow. Simultaneous or subsequent reorientation of the acetabulum by triple TPO (Pediatric Triple Osteotomy) or PAO (Peri-Acetabular Osteotomy) may be necessary.

**Indications:**

Surgical hip dislocation with femoral head reduction can be performed at any age in cases with hinge abduction and Stulberg class IV and V deformity. Procedure indicated for patients with active or healed disease. After the resection, a viable residual femoral head must remain, i.e. at least 50% of the expanded femoral head, which is best planned using “comparative” 3D reconstruction.

**Contraindications:**

Completely destroyed cartilage or femoral head.

**Surgical technique:**

The same surgical procedure as described for classic surgical hip dislocation is followed. Preparation of retinacular flaps. With detailed knowledge of the vascular supply and precise execution of this technique, blood supply to the femoral head will be preserved; once safely surgically dislocated, the femoral head and neck can be split and the necrotic part of the femoral head removed. Reformation of the femoral head as spherical as possible is achieved by screw fixation of the femoral neck to align the two articular parts of the femoral head. Distalization and fixation of the great trochanter helps to restore offset (functional femoral neck length). Depending on the congruence and stability of the femoral head in the acetabulum, a primary TPO or PAO may also be necessary.

**Postoperative management:**

Intraoperative stability must be achieved to ensure functional posttreatment without a hip spica cast. Walking with crutches with toe contact only is advised. Active rotation is not allowed. Active and passive flexion up to 90° allowed. These measures have to be observed for 8–10 weeks. Then, active physiotherapy rehabilitation may commence, depending on healing, as assessed clinically and radiologically.

**Results:**

Our published follow-up examinations (currently 21 years) show consistently good results with a technically correct operation and correct indication as well as adequate follow-up treatment. No necrosis of the reduced femoral head has been observed. All split femoral heads and femoral necks are primarily healed.

## Vorbemerkungen

Der Verlauf eines Morbus Perthes ist extrem variabel, von kaum sichtbaren Veränderungen des Femurkopfes bis hin zu schwersten Deformitäten und steifen Hüftgelenken. Dabei ist zu beobachten, dass die Symptomatik nicht immer synchron zur Schwere und der zu erwartenden Kopfdeformität einhergeht. Wir gehen davon aus, dass sehr viele Erkrankungen „stumm“ verlaufen und unter Umständen wegen Früharthrose der Hüfte erst im Alter von 30 oder 40 Jahren retrospektiv diagnostiziert werden [6, 19].

Lange Zeit hat man sich nur auf die von Catterall [2, 4] erstellten Stadieneinteilung verlassen, um die Schwere des Verlaufes einzuschätzen, später kamen andere Einteilungen wie die „Lateral Pillar“-Klassifikation [1] und andere dazu. Auf der anderen Seite haben Stulberg et al. eine Klassifikation geschaffen, die „post festum“ das Ausheilungsergebnis beschreibt [4, 12].

Zwischenzeitlich hat sich eine differente Betrachtungsweise des Morbus-Perthes-Verlaufes eingestellt. Es wird mehr Wert auf die Entwicklung/Beziehung der Stellung zwischen Hüftkopf und Hüftpfanne gelegt. Das große Schlagwort heißt dabei „Containment“ und beschreibt die Relation zwischen dem Hüftkopf, praktisch unabhängig der Schwere des Kopfbefalles, und der Hüftpfanne. Solange diese Relation stimmt und der Hüftkopf ein perfektes Containment zeigt, ist im Prinzip keine spezifische Therapie notwendig, zeigt sich jedoch im Verlauf, auch wenn der Hüftkopf nur gering befallen ist, dass dieses Containment nur minimal verloren geht, sollte eine Containment-verbessernde Operation im Sinne einer Triple Osteotomie/PAO oder milden intertrochantären Osteotomie angestrebt werden [6, 15]. Eine Salter-Osteotomie empfehlen wir wegen der zusätzlichen Lateralisation, Druckerhöhung und der Gefahr der Retroversion nicht.

Da sich einerseits diese Denkweise der optimalen Containment-Erhaltung noch nicht flächendeckend durchgesetzt hat, sehen wir im Früh- wie auch dann im Spätstadium schwere Kopfdeformierungen, die man sicherlich hätte vermeiden können. Auf der anderen Seite, wie oben beschrieben, gibt es sehr stille Verläufe, wo die Symptomatik erst in einem späten Stadium aufgrund von schon vorhandenen Gelenkschäden auftritt. Der Femurkopf hat dabei eine typische, asphärische, inkongruente Form (als Stulberg IV und V bezeichnet) und ist oft auch massiv vergrößert, dafür jedoch abgeflacht. Er weist die typische „Champignon“-Form auf, wobei der Kopfrand meist den Schenkelhals überragt. In der Literatur wird dafür der Term „coxa magna“ oder „coxa plana“ verwendet. Der vergrößerte und abgeflachte Kopf verliert sein Containment und lateralisiert sich. Dabei kommt es zum typischen Impingement und progressiver Zerstörung des Labrums bis hin zum knöchernen, lateralen Pfannenrand [6, 19, 21]. Passiert dieser Prozess in einem frühen Stadium der Krankheit, resultiert durch Abduktion der Hüfte ein „zweihöckriger“ Hüftkopf als sog. „Kamelbuckel-Form“. Funktionell resultiert die in der Literatur beschriebene „hinged abduction“, welche dazu führt, dass der Femurkopf progredient bei jeder Abduktion aus der Pfanne gehebelt wird ([22]; vgl. Abb. 2). Die Delle zwischen den beiden Höckern entspricht auch meist der Region mit der ausgeprägtesten Nekrose.

In der Vergangenheit wurde versucht, dieses Problem durch augmentierende Operationen wie die Chiari Osteotomy oder Shelf-Operationen zu behandeln. Dabei konnte jedoch die Kopfdeformität nie adressiert werden, sondern die Operation bestand darin, den extraartikulären Kopfanteil zu überdachen. Die Sphärizität wurde nicht verbessert. Zudem hat man versucht, mehrheitlich im Frühstadium, durch eine varisierende proximale Femurosteotomie den Hüftkopf wiederum ins Acetabulum einzustellen, was prinzipiell nur möglich ist, wenn die Abduktionsaufnahme eine Reposition der Hüfte zeigt. Definitionsgemäß ist dies jedoch bei einem 2‑höckrigen Hüftkopf mit „hinged abduction“ nicht möglich.

Der direkte Einblick in die Hüfte respektive auf den vom Perthes veränderten Hüftkopf aufgrund einer heute als sicher geltenden operativen Methode zur Luxation des Hüftkopfes [5] hat einerseits das Verständnis um die Veränderungen an der Hüfte in vielen Belangen verändert und verbessert, andererseits aber auch den Weg zu einem aktiven chirurgischen Vorgehen direkt am Ort der Veränderung ermöglicht – dies im Gegensatz zu all den bisherigen, bekannten operativen Verfahren am proximalen Femur oder an der Hüftpfanne, die fernab der eigentlichen Deformität des Hüftkopfes korrigieren und in den meisten Fällen in sekundären, durch die Operation bedingten Veränderungen enden, wie z. B. groteske Varusstellungen nach intertrochantärer Osteotomie [3]. Die Erfahrungen der ersten 21 Jahre sind sehr Erfolg versprechend und zeigen zumindest über diese Zeitperiode bei allen Patienten eine sehr gute Funktion und Hüftsituation, die einen allfälligen hüftprothetischen Ersatz deutlich in ein späteres Alter zu verschieben mag [8–11, 16, 17].

Um eine sichere Femur-Kopf-Verkleinerungsoperation vornehmen zu können, ist die Kenntnis der Blutversorgung am proximalen koxigialen Femurende [7, 8] ebenso notwendig wie die Beherrschung der schonenden und sicheren chirurgischen Hüftluxation [14]. Im vorliegenden Artikel sollen deshalb nur die wesentlichsten Schritte der chirurgischen Hüftluxation dargestellt werden. Das Hauptaugenmerk ist dabei auf die Präparation der reticulären Flaps und der eigentlichen Kopfverkleinerung gerichtet. Zum Studium der Präparation der retinakulären Flaps wie der chirurgischen Hüftluxation sei folgende Literatur empfohlen: [5, 8, 11, 16, 20].

## Operationsprinzip und -ziel

**Figure 1:**
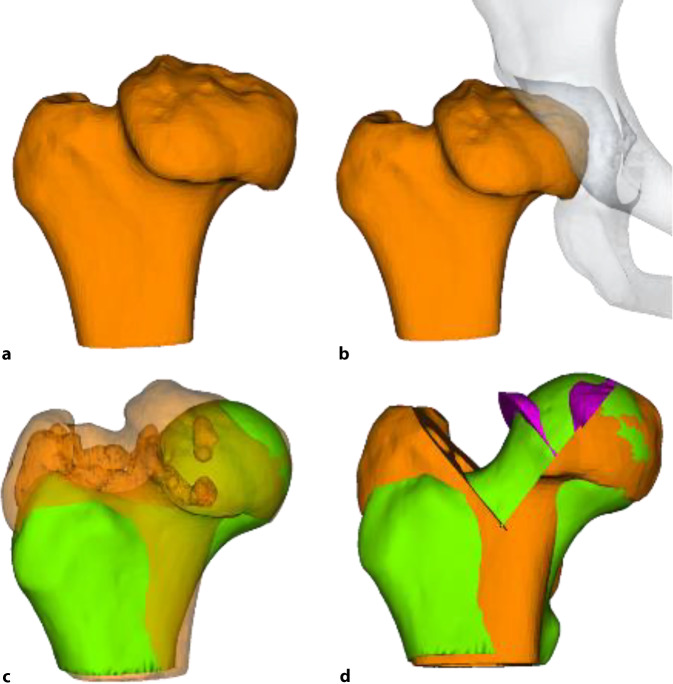

Das Operationsprinzip besteht darin, dass man über eine heute als sicher durchführbar und somit als sicher geltende chirurgische Hüftluxation den vollen 3‑dimensionalen Überblick auf den veränderten Hüftkopf bekommt [5]. Anhand der vorgängig durchgeführten hochauflösenden MRI-Untersuchung sowie der vor Ort sich darstellenden Situation wird eine zentrale Hüftkopfresektion vorgenommen, um eine bestmögliche Sphärizität zu erreichen. Die Schnittführung für eine optimale Sphärizität kann heute auch vorgängig aufgrund einer 3‑D-CT-Planung (Abb. 1a–c) vorgenommen werden, da diese Planung einen Vergleich mit der gesunden Seite erlaubt (Abb. 1d). Man muss sich jedoch bewusst sein, dass dies eine „theoretische“ Planung ist und allfällig wegen der Gefäßversorgung nicht genau so durchgeführt werden kann. Deshalb sei nochmals betont, dass die Kenntnis um die Gefäßversorgung des Hüftkopfs absolute Voraussetzung für diese Operation ist.

Es kann kaum genügend betont werden, wie wichtig es ist, die sog. retinakulären Flaps zu bilden, um die Blutversorgung der verbleibenden Kopfanteile sicherzustellen [8]. Schlussendlich ist das Operationsziel, eine möglichst optimale Kongruenz/Containment der Hüfte zu erreichen mit einer bestmöglichen Restfunktion. Es ist evident, und es wäre illusorisch zu behaupten, dass man auf jeden Fall eine normale Hüftfunktion erreichen kann. Diese Operation soll dem Patienten bei fehlenden anderen äquivalenten Möglichkeiten erlauben, über eine Periode von 20 bis 30 Jahren einer normalen täglichen Aktivität nachzugehen, ohne dabei von Schmerzen geplagt zu werden. Dass dieses Ziel erreicht werden kann, zeigen die ersten Resultate nach 21 Jahren.

### Vorteile

- Bestmögliche Wiederherstellung der Hüftkopfsphärizität
- Verbesserung der Funktion
- Reduktion der Früharthrose

### Nachteile

- Gefahr der zusätzlichen Verschlechterung der Hüftkopfzirkulation durch unsachgemäßer chirurgischer Hüftluxation
- Heilungsstörungen
- Erfordert fundierte hüftchirurgische Kenntnisse
- Im Falle einer verbliebenen Incongruenz infolge eines „ausgeweiteten Acetabulum“ sollte dies erkannt und durch eine zusätzliche PAO oder Triple OT behoben werden.

## Indikationen

Die Indikationsstellung richtet sich im Wesentlich nach den nachfolgend aufgeführten Punkten. Im Einzelnen müssen diese sehr gut abgewogen, auf alternative Verfahren geprüft und diskutiert werden. „Harte“ Fakten existieren im Moment noch nicht. Wichtig scheint uns, dass sicherlich noch 50 % des ursprünglich deformierten Kopfes nach der Resektion einen noch tragfähigen Hüftkopf bilden können. Um diese Abschätzung bereits präoperativ machen zu können, wird sich in Zukunft sicherlich die vergleichende 3‑D-Darstellung eignen (nicht berücksichtigt wird hier die Gefäßversorgung).

Der Zustand des Knorpels im zu entfernenden Kopfanteil sowie die Tiefe der Nekrose spielen dabei keine Rolle. Obwohl eine obligate MRT-Untersuchung gute Anhaltspunkte über den Kopf-/respektive Knorpelzustand geben kann, zeigt doch die klinische Erfahrung, dass die abschließende Beurteilung nur bei luxierter Hüfte gemacht werden kann. Der in Abb. 3 dargestellte Kopf zeigte sich im MRT als stabil in den Randzonen, intraoperativ jedoch war eine Rekonstruktion im Sinne der Kopfreduktion nicht möglich. Hier wurde ein Kopfaufbau mit trikortikalem Knochenblock durchgeführt.

**Figure 2:**
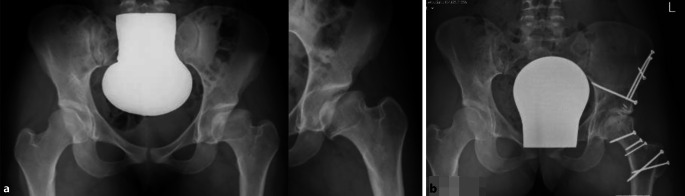

- Jegliche Inkongruenz, Asphärizität des Hüftkopfes praktisch unabhängig vom Alter (Stuhlberg IV und V) wie in Abb. 2a ersichtlich ist
- „Hinged abduction“
- Noch aktiver oder bereits abgeschlossener Morbus Perthes

## Kontraindikationen

**Figure 3:**
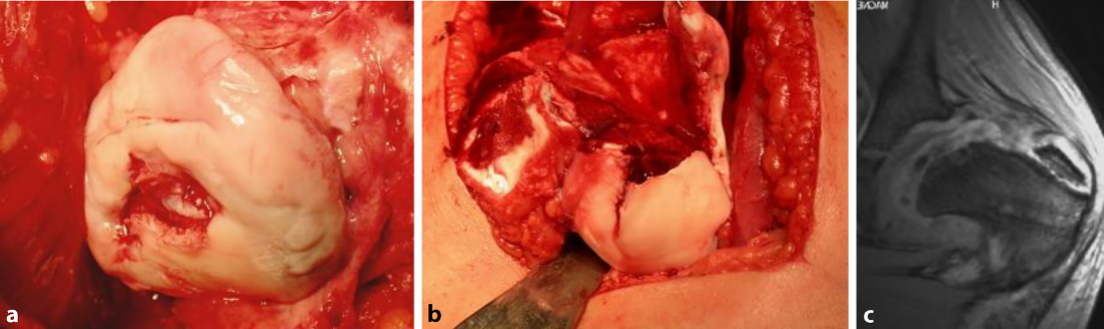

- Völlig zerstörter Hüftkopf (Abb. 3)
- Kinder unter 6 bis 8 Jahren (nur bedingte KI)

## Patientenaufklärung

- Offene und umfassende Aufklärung der Eltern/Patienten über alle möglichen Behandlungsverfahren zur Behandlung von Komplikationen beim Morbus Perthes
- Begründung, weshalb in der vorliegenden Situation dieses Verfahren gewählt wird
- Allgemeine Operationsrisiken
- Spezifische Risiken dieser Operation wie: Nichtverheilen der beiden zusammengefügten Kopfanteile, Nekrose der verbleibenden Kopfanteile, verzögerte Heilung der Trochanterosteotomie
- Allfällige Erweiterung des Eingriffes im Sinne einer PAO respektive Triple-OT
- Mögliche Zweitoperation im Falle einer Hüftinstabilität, die initial nicht so eingeschätzt wurde
- Mögliche residuelle Fehlstellungen oder Fehlfunktionen
- Diskussion, weshalb ein Hüftgelenkersatz in diesem Alter nicht als sinnvolle Alternative gesehen werden kann
- Angebot einer krankenhausexternen Pflege
- Heilungsdauer
- Physiotherapie

## Operationsvorbereitungen

- Röntgen Becken a.-p. sowie Abduktionsaufnahme zur Verifizierung der „hinged abduction“
- MRT (wenn immer möglich Arthro-MRT und radiäre Sequenzen) zur Beurteilung des Nekroseausmaßes
- Bildanalyse und evtl. Studium zusätzlicher bildgebender Verfahren
- Zumindest skizzenhafte Planung der möglichen Schnittführung durch den Femurkopf und Ausmaß der Reduktion, am besten anhand der speziellen radiären MRT-Sequenzen
- CT- und 3‑D-Darstellung nicht obligat, kann im Einzelfall hilfreich sein zur Planung der Resektionsflächen (noch in der experimentellen Phase der Umsetzung)
- Lagerungsmaterial bereitstellen
- Möglichkeit der intraoperativen Durchleuchtung prüfen
- Besprechung mit der Anästhesie bezüglich Relaxation, Blutverlust und Operationsdauer; allfällig Einsatz eines „Cell Saver“
- Orientierung/Besprechung dieses komplexen Eingriffes mit dem OP-Personal

## Instrumentarium

**Figure 4:**
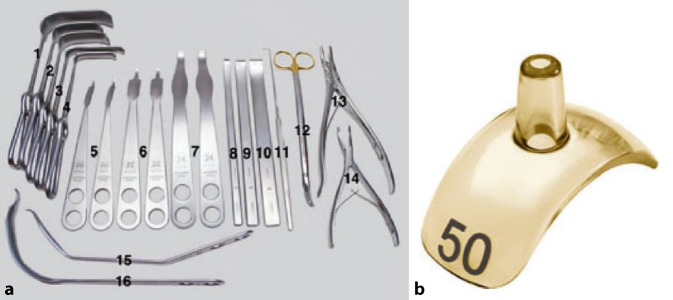

Bei diesem doch sehr spezifischen und komplexen Eingriff ist das richtige Instrumentarium von entscheidender Wichtigkeit; dies wird sehr oft unterschätzt. Wir empfehlen das von uns entworfene und über spezialisierte Instrumentenfirmen^1^ vertriebene Instrumentenset. Dieses sollte als Minimum folgende Instrumente enthalten:

- verschieden breite und hohe Wundretraktoren mit abgerundeten Kanten (Breite 14 mm, 25 mm, 30 mm; Höhe 55 mm, 70 mm, 100 mm), alle in zweifacher Ausführung
- Je 2 Hohmann Haken schmal 8 mm und breit 18 mm
- Je 2 große und kleine, sog. „reversed“ abgerundete Hohmann-Haken
- 240 mm lange schmale (10 mm), mittlere (15 mm) und breite (20 mm) Lambotte (Simal)-Meißel gerundet
- Nervenhäkchen lang
- Lange, stark gekurvte Schere (aus Gynäkologie bekannt)
- Zwei verschieden große und scharfe Rongeur-Zangen
- Kräftiger 1‑Zinker-Haken zum Hochheben des Femurs bei der Luxation
- Doppelt gekrümmter langer Hohmann-Haken (bei uns Giraffe genannt)
- Lang gebogener, breiter Hohmann-Haken (auch Easy Rider genannt)
- FAI (Femoral Template) zur Überprüfung der Grösse und Sphärizität (42–58 mm) des zerstörten Hüftkopfes (Abb. 4)

## Anästhesie und Lagerung

**Figure 5:**
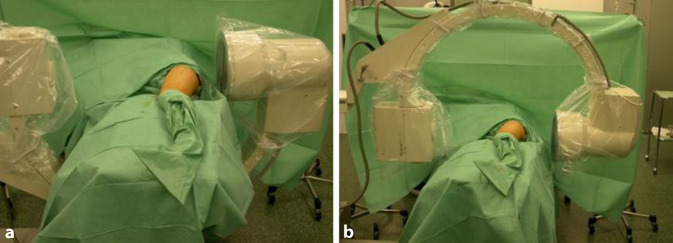

- Intubationsnarkose; Relaxation ist Voraussetzung und erleichtert die Präparation und Luxation
- Epiduralkatheter zur postoperativen Schmerztherapie
- Je nach Durchleuchtungstechnik
  - C‑Arm so positionieren, dass er über den Patienten gefahren werden kann oder
  - unter dem Operationstisch durch. Diese Positionierung lässt aus Sterilitätsgründen (die unter dem Operationstisch nicht gegeben ist) weniger Manipulationen des C‑Armes zu (Abb. 5).
- Wir bevorzugen wegen des besseren Bildes, des größeren Bildausschnittes, der genaueren Durchleuchtungspositionierung und der dadurch kürzeren Durchleuchtungszeit, dass die Bildplatte des Durchleuchtungsgerätes direkt von dorsal am Patienten anliegt (Abb. 5).
- Desinfektion und Abdeckung des ganzen Beines bis auf Höhe des Nabels. Der Unterschenkel bis Mitte Oberschenkel wird dann zusätzlich steril eingepackt.
- Anbringen eines sterilen Sackes auf der ventralen Operationstischseite des Patienten zwecks steriler Lagerung des Unterschenkels nach der Hüftluxation [5].

## Operationstechnik

Abb. 6, 7, 8, 9, 10, 11, 12, 13, 14, 15, 16, 17, 18, 19 und 20.

### Schritt 1: Lagerung und chirurgische Hüftluxation

**Figure 6:**
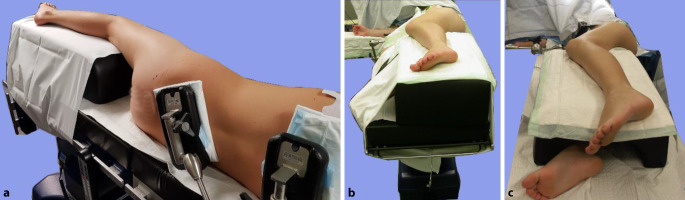

**Figure 7:**
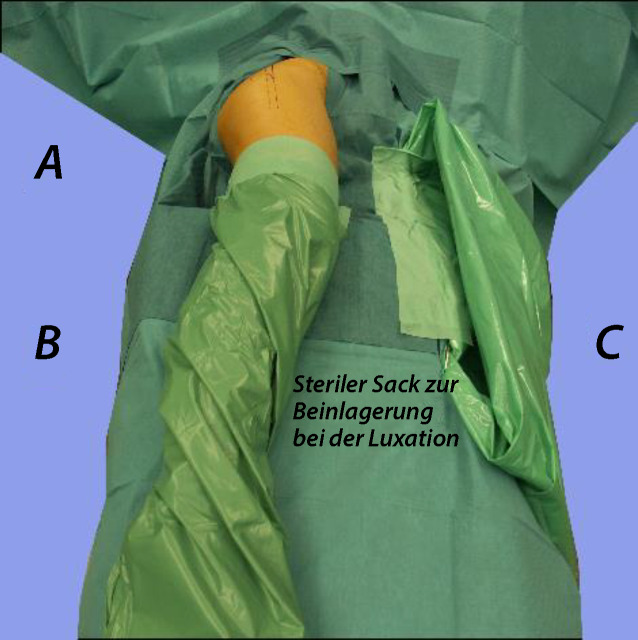

**Figure 8:**
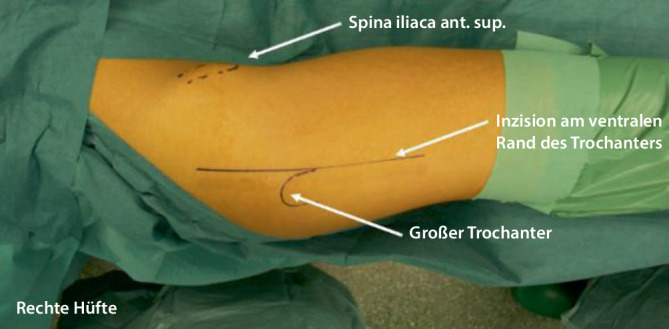

**Figure 9:**
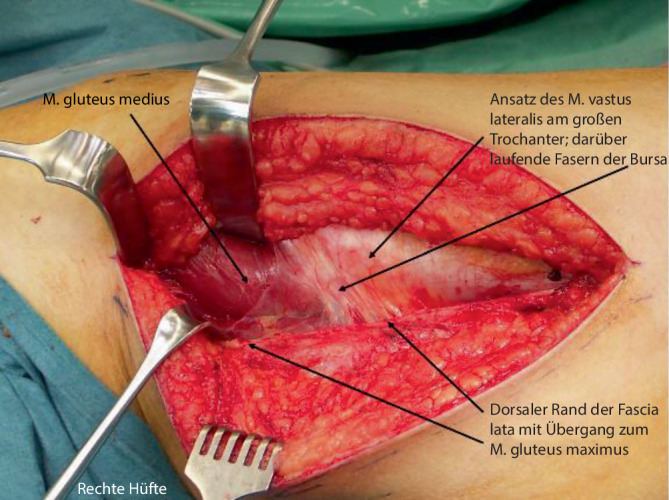

**Figure 10:**
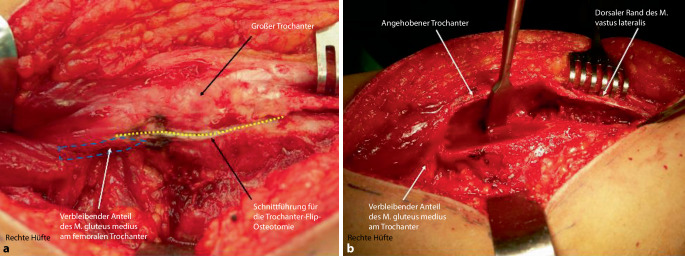

**Figure 11:**
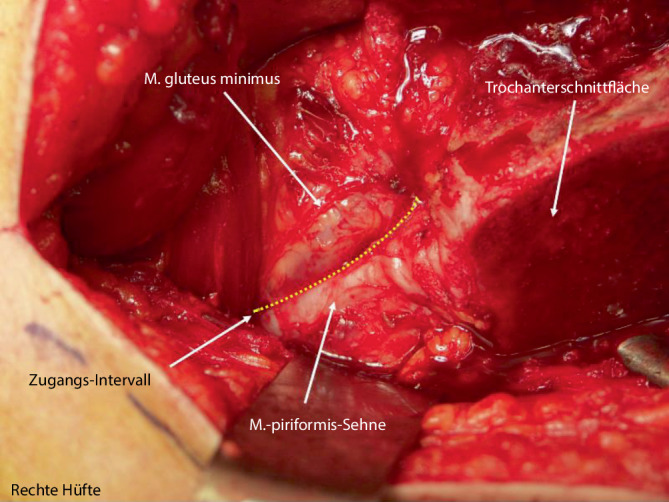

Abb. 6, 7, 8, 9, 10, 11, 12 und 13.

**Figure 12:**
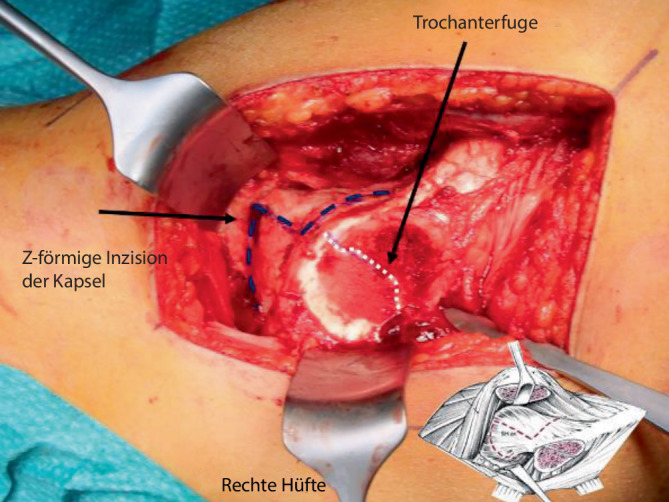

**Figure 13:**
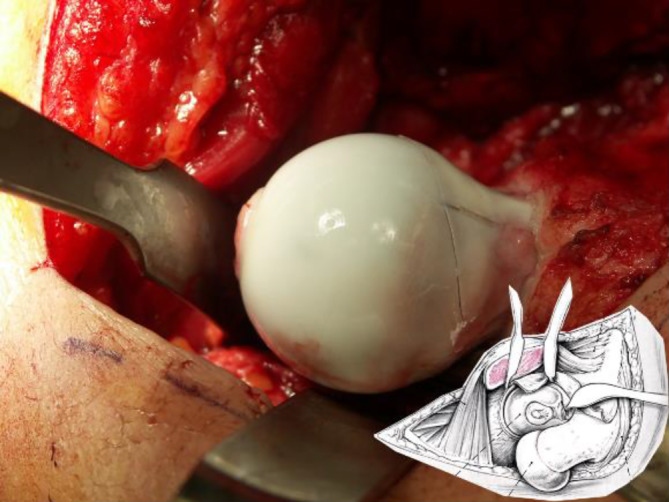

### Schritt 2: Präparation der retinakulären Flaps

**Figure 14:**
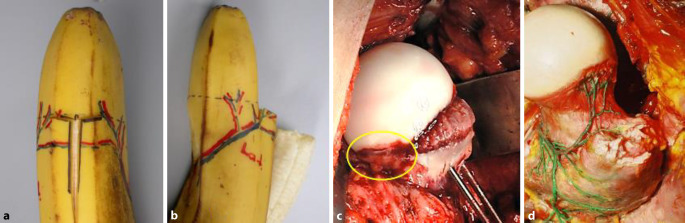

**Figure 15:**
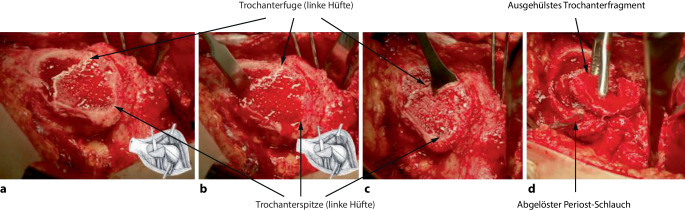

Abb. 14, 15, 16 und 17.

**Figure 16:**
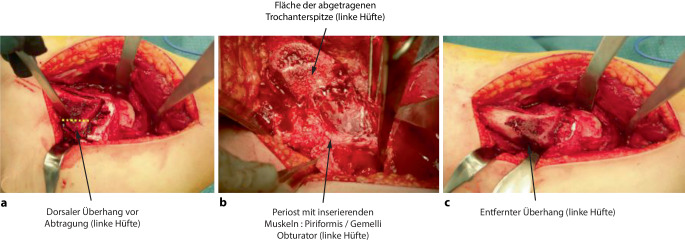

**Figure 17:**
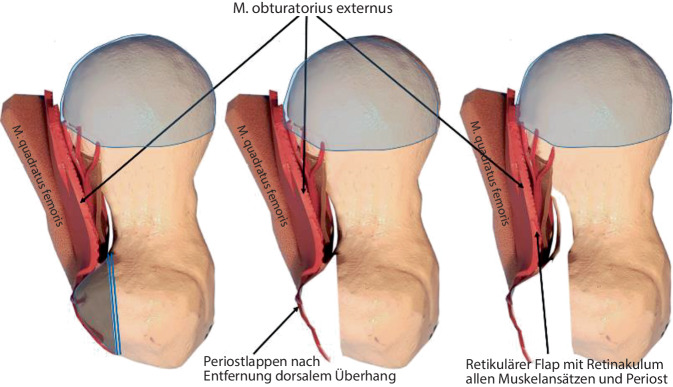

### Schritt 3: Hüftkopfreduktionsplastik

**Figure 18:**
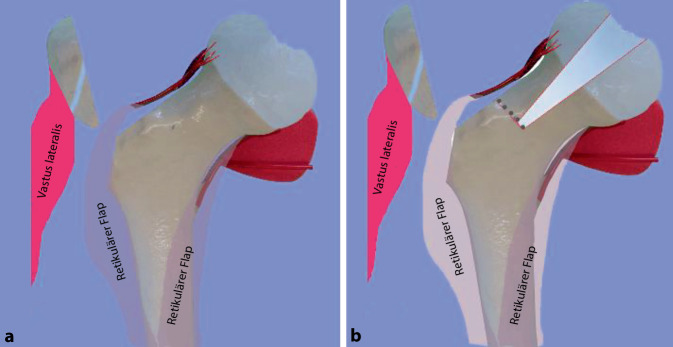

Die Abb. 18, 19 und 20 zeigen die einzelnen Schritte anhand des von uns anhand von CT-Bildern angefertigten Knochenmodelles.

**Figure 19:**
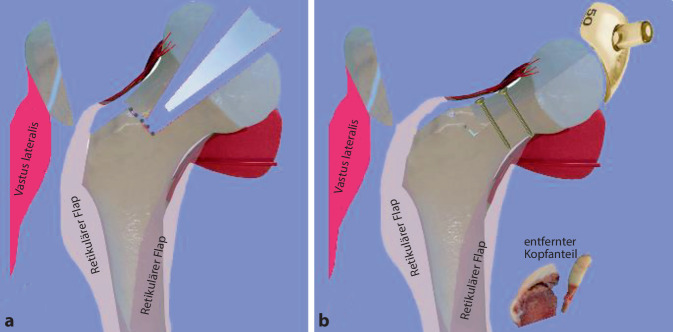

**Figure 20:**
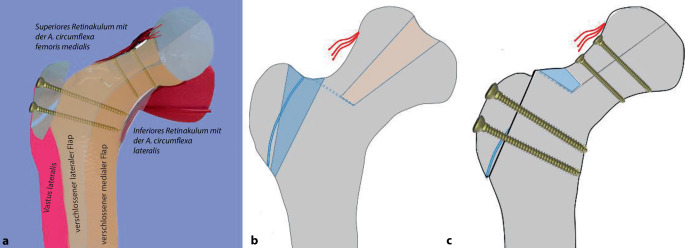

### Besonderheiten

Nach stabiler Verschraubung der Kopfanteile und Readaptation der beiden Flaps/Retinacula + Gefäße erfolgt die behutsame Reposition der Hüfte. Ein Einklemmen der Gefäße muss vermieden werden … Oft ist aufgrund der Kopfdeformität das Acetabulum „ausgeweitet“ und der Hüftkopf steht nicht stabil in der Pfanne, oder wir haben ein extremes Missverhältnis zwischen Kopf- und Hüftpfannenradius. Es ist wichtig, sich dieses Umstandes bewusst zu sein und zu entscheiden, brauche ich eine stabilisierende Zusatzoperation im Sinne einer Triple-OT oder PAO, oder kann ich zuwarten. Wie von uns in der Literatur beschrieben [16–18], ist dieser Entscheid nicht immer einfach, die Stabilität sollte aber immer angestrebt werden. Es ist auch wichtig, dass im Vorfeld der Operation der Patient respektive die Eltern darüber in zweierlei Hinsicht orientiert worden sind; erstens, dass es im Entscheid des Operateurs liegen muss, ob gleichzeitig eine Pfannen-Schwenk-Operation gemacht wird, oder zweitens, dass es sein kann, dass innerhalb von Monaten eine solche Operation durchgeführt werden muss, sollte sich zeigen, dass die Hüfte nicht stabil ist. In unserem Krankengut, das inzwischen größer ist als die publizierten Fälle, zusätzlich die noch in Drittkliniken versorgten Fälle haben wir in etwa folgende Situation: 40 % brauchen keine zusätzliche azetabuläre Korrektur, weder initial noch später, ca. 30 % brauchen initial eine Triple oder PAO und 30 % innerhalb des ersten Jahres. Bei diesen Zahlen besteht die berechtigte Frage, weshalb man nicht bei allen unsicheren Fällen gleich zur Pfannenoperation übergeht. Dieser Entscheid ist nicht nur aus ärztlicher Sicht zu sehen, sondern wird auch durch die Ansprüche, Wünsche des Patienten mitbeeinflusst. Tendenziell möchte der Patient so wenig wie möglich operiert haben, obwohl von der Sache her doch eine umfassende Operation angezeigt wäre. Es ist dann das Geschick und die Kompetenz des Chirurgen, den Patienten von der Notwendigkeit eines kombinierten Eingriffs zu überzeugen.

Im Weiteren ist ebenfalls wichtig zu erklären, dass nach einer solchen Operation die Hüfte niemals „normal“ sein kann; Ziel ist es, die initial schwerstens veränderte Hüfte für einen möglichst langen Zeitraum schmerzfrei und funktionsfähig zu halten. Aktuell überblicken wir einen Zeitraum von 21 Jahren und können feststellen, dass weit über 80 % der Patienten keine weitere Operation brauchten.

Sicher ist auch zu diskutieren, was man bei völlig zerstörtem Hüftkopf, der sich jedoch im MRT nicht so dargestellt hat (Abb. 3), als intraoperativen Plan B hat. Für uns ist bis heute der Einbau einer Hüfttotalprothese keine Option; vielmehr würden wir in dieser Situation auf eine Arthrodese ausweichen. Der Hauptgrund gegen TP in diesem Alter ist das Überstrapazieren der TP durch den jugendlichen Übermut und Aktivität! Die Arthrodese kann dann im höheren Alter, falls notwendig, in eine TP umgewandelt werden. Die Diskussion über dieses Vorgehen ist sicherlich noch offen, ist aber in unserer Praxis der adäquateste Schritt. Zudem wird diese Option im Vorfeld mit dem Patienten respektive den Eltern immer diskutiert.

## Postoperative Behandlung

Prinzipiell unterscheidet sich die postoperative Behandlung nicht wesentlich von anderen hüfterhaltenden Operationen wie Impingement, SCFE oder relativer SH-Verlängerung oder selbst nur intertrochantären Korrekturen.

- Radiologische Kontrolle mit Becken a.‑p. unmittelbar postoperativ sowie nach 6 bis 8 Wochen zur Konsolidations- und Stellungskontrolle
- Mobilisation in der Sagittalebene Flexion/Extension bis 60°, vorzugsweise mit CPM
- Stockentlastung mit Bodenkontakt
- Nach sicherer Konsolidation Übergang zur Vollbelastung und Beginn mit rotatorischen Übungen und Krankengymnastik
- Sportverbot für mindestens 3 Monate
- Eine routinemäßige Thromboseprophylaxe sehen wir aufgrund der fehlenden Evidenz und der eigenen Erfahrung im Kindes- und Jugendalter nicht als indiziert; Ausnahmen bilden Übergewicht, rauchen, Antikonzeption, familiäre Belastung

## Fehler, Gefahren, Komplikationen

- Der größte Fehler besteht darin, ohne genügend Erfahrung oder Beiziehen eines erfahrenen Kollegen, sich an eine solche Operation zu wagen!
- Verursachen einer AVN als schwere Komplikation schon rein durch den Zugang zur Hüfte, der lediglich als eigentliche Vorbereitung der Chirurgie am Hüftkopf gilt
- Fehlende Kenntnis der retikulären Flap-Präparation
- Trotz Femurkopfverkleinerung keine genügende Reposition → Überprüfen der azetabulären Situation, allfällig zusätzlich Pfannenschwenkung
- Non-Union des Trochanter major → bessere Fixierung
- Schädigung des N. ischiadicus durch ungenügend ausgedehnte Präparation für die chirurgische Hüftluxation
- Heterotope Ossifikationen (beim Jugendlichen selten)
- Intraartikuläre Verwachsungen mit massiver Bewegungseinschränkung → allfällig arthroskopische Revision und Adhäsiolyse
- Trotz optimaler Operation rasch fortschreitende Verschlechterung der Hüftsituation; möglicher Grund: falsch eingeschätzte, bereits zu fortgeschrittene Knorpelzerstörung respektive auf azetabulärer Seite Delamination (auch als Carpet-Phänomen bezeichnet) des Knorpels

## Ergebnisse

Unsere Erfahrungen mit der Hüftkopfreduktionsplastik über eine chirurgische Hüftluxation sind über die letzten 15 Jahre sehr positiv. Wie unseren in dieser Zeit durchgeführten Publikationen [8, 11, 16, 17] zu entnehmen ist, haben wir keine chirurgisch-technischen Komplikationen wie auch keine Nekrose des freien, nur noch am superioren Retinaculum hängenden Kopfanteiles gesehen. Dies ist sicherlich auch darauf zurückzuführen, dass diese Operationen praktisch nur durch 4 eng miteinander zusammenarbeitende Chirurgen durchgeführt wurde. Auch von den zahlreichen als Teaching-Operation durchgeführten Eingriffen in Drittkliniken kamen nur positive Rückmeldungen. Dennoch darf der Erfolg dieser Operationstechnik nicht überbewertet werden, und weitere Langzeitverläufe müssen deren zumindest mittelfristig positiven Resultate bestätigen. Als mittelfristig sehen wir eine Verbesserung zumindest über 15 bis 20 Jahre an. Aktuell ist in 1 Falle nach 10 Jahren eine rapide Verschlechterung im Sinne einer schweren Arthrose aufgetreten, obwohl das Containment perfekt war. Hier konnte jedoch durch intertrochantäre Umstellung zumindest kurzfristig die Situation wiederum verbessert werden.

Der hier dargestellte Verlauf dokumentiert die Wichtigkeit, initial die Schwere der Femur-Kopfschädigung einerseits und der Zerstörung des Kopfknorpels richtig einzuschätzen. Ein solches Beispiel ist in Abb. 3 dargestellt. Obwohl MRT-mäßig hätte vermutet werden können, die Situation durch eine Kopfverkleinerung verbessern zu können, hatte sich intraoperativ gezeigt, dass dies in diesem Fall nicht zielführend gewesen wäre.

### Klinische Fallbeispiele

Darstellung von 3 typischen Fallbeispielen entsprechend den verschiedenen Optionen:

1. nur Kopfreduktion,
2. Kopfreduktion und gleichzeitig Pfannenschwenkung,
3. Kopfreduktion und sekundäre Pfannenschwenkung.

#### Fall 1: alleinige Kopfreduktion

Abb. 21, 22 und 23.

**Figure 21:**
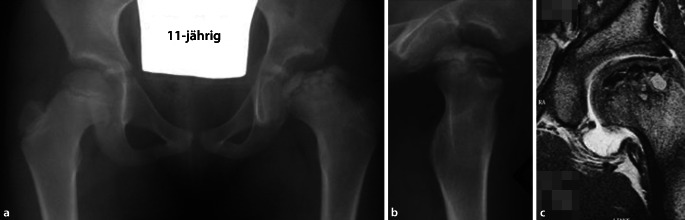

**Figure 22:**
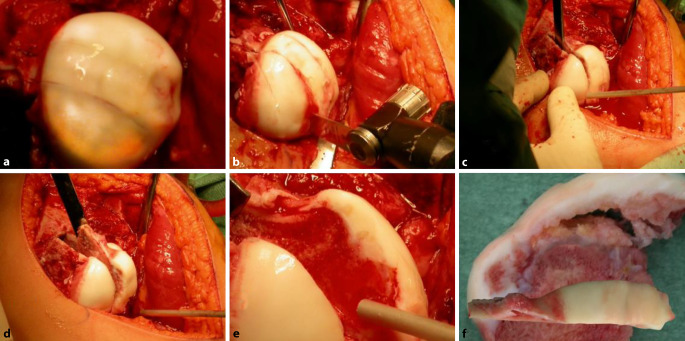

**Figure 23:**
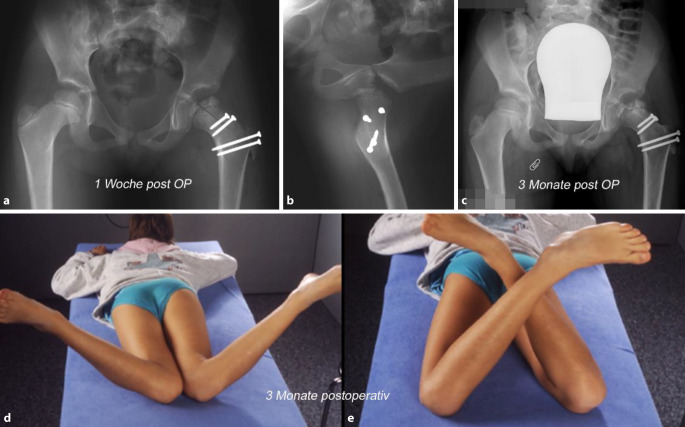

#### Fall 2. Kopfreduktion und gleichzeitige Triple-Osteotomie, Operation im Alter von 10 ½ Jahren

**Figure 24:**
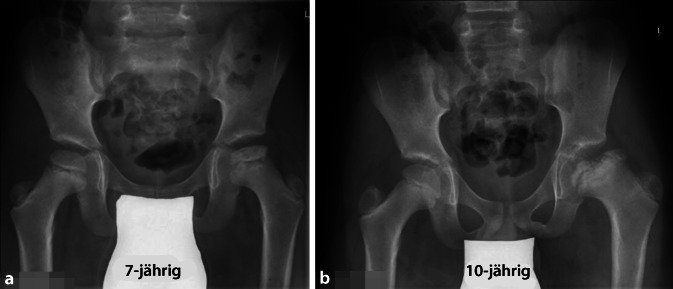

**Figure 25:**
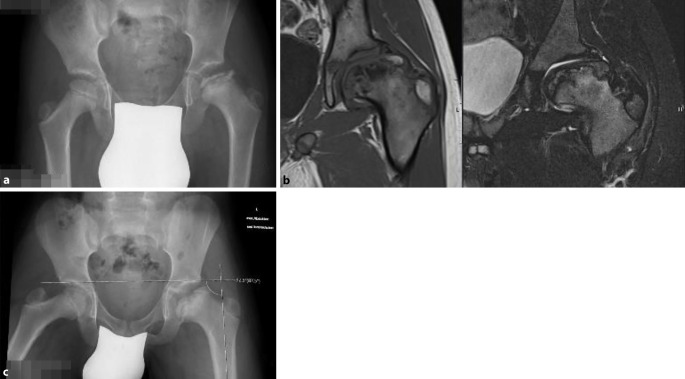

**Figure 26:**
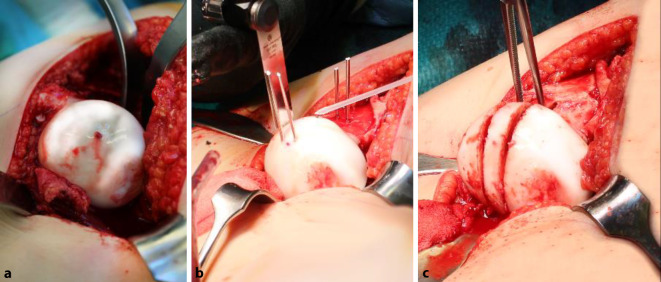

**Figure 27:**
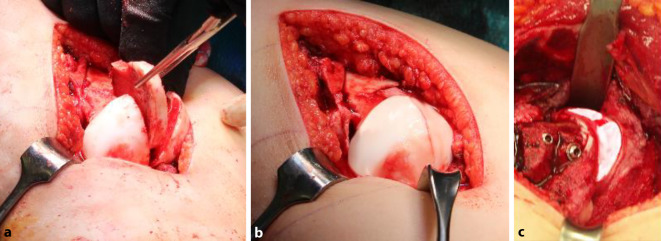

Abb. 24, 25, 26, 27, 28 und 29.

**Figure 28:**
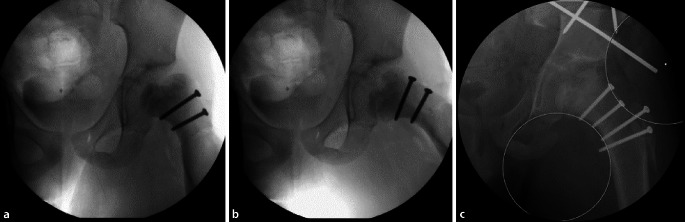

**Figure 29:**
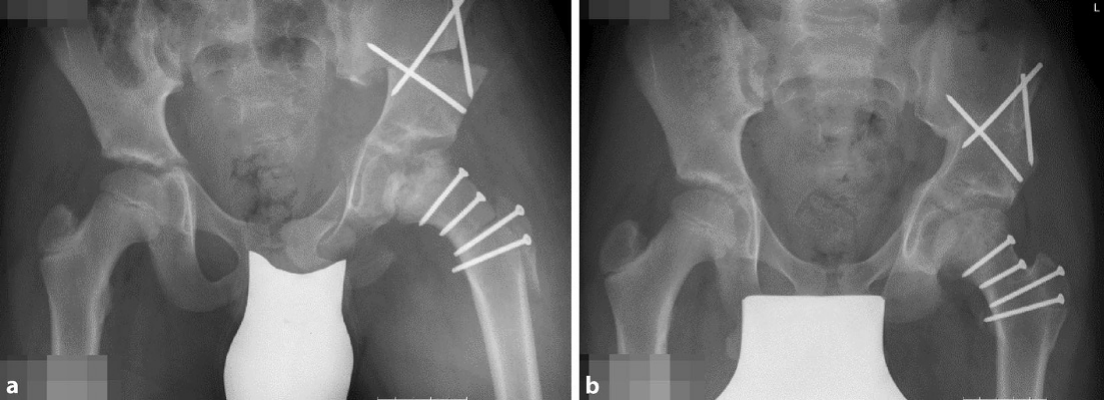

#### Fall 3: Hüftkopfreduktion und aufgeschobene Triple-Osteotomie

Abb. 30 und 31.

**Figure 30:**
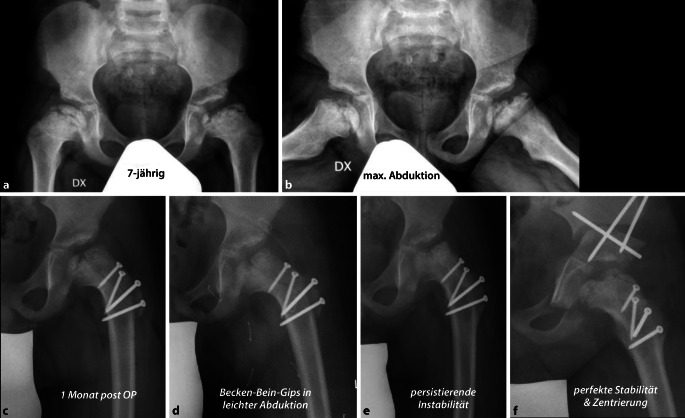

**Figure 31:**
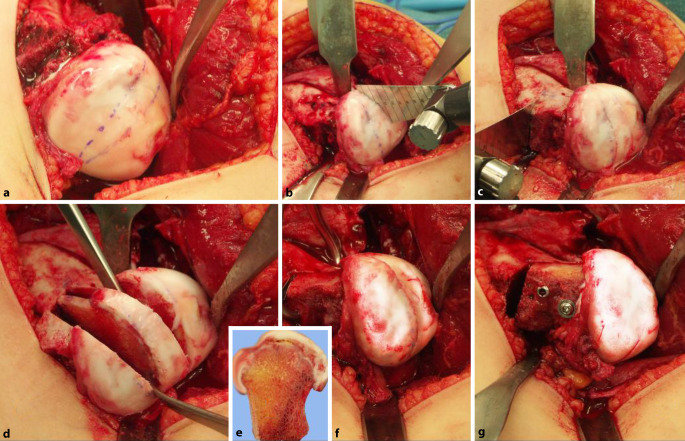

## Footnotes

1 **Manufaktur; **Accuratus AG, Holligenstrasse 94, 3008 Bern, Switzerland. **Referenzen:** https://subtilis.ch/referenzen.html, mit freundl. Genehmigung von Accurates AG, Bern.
